# Comparative Effectiveness of Microdecompression Alone vs Decompression Plus Instrumented Fusion in Lumbar Degenerative Spondylolisthesis

**DOI:** 10.1001/jamanetworkopen.2020.15015

**Published:** 2020-09-10

**Authors:** Ivar Magne Austevoll, Rolf Gjestad, Tore Solberg, Kjersti Storheim, Jens Ivar Brox, Erland Hermansen, Frode Rekeland, Kari Indrekvam, Christian Hellum

**Affiliations:** 1 Kysthospitalet in Hagevik, Orthopedic Clinic, Haukeland University Hospital, Bergen, Norway; 2 Department of Clinical Medicine, University of Bergen, Bergen, Norway; 3 The Norwegian Registry for Spine Surgery, University Hospital of Northern Norway, Tromsø, Norway; 4 Research Department, Division of Psychiatry, Haukeland University Hospital, Bergen, Norway; 5 Institute of Clinical Medicine, Arctic University of Norway, Tromsø, Norway; 6 Research and Communication Unit for Musculoskeletal Health, Oslo University Hospital, Oslo, Norway; 7 Department of Physical Medicine and Rehabilitation, Oslo, University Hospital, Oslo, Norway; 8 Department of Orthopedic Surgery, Ålesund Hospital, Møre and Romsdal Hospital Trust, Ålesund, Norway; 9 Division of Orthopaedic Surgery, Oslo University Hospital, Oslo, Norway

## Abstract

**Importance:**

Conflicting evidence and large practice variation are present in the surgical treatment of degenerative spondylolisthesis. More than 90% of surgical procedures in the United States include instrumented fusion compared with 50% or less in other countries.

**Objective:**

To evaluate whether the effectiveness of microdecompression alone is noninferior to decompression with instrumented fusion in a real-world setting.

**Design, Setting, and Participants:**

This multicenter comparative effectiveness study with a noninferiority design assessed prospective data from the Norwegian Registry for Spine Surgery. From September 19, 2007, to December 21, 2015, 1376 patients at 35 Norwegian orthopedic and neurosurgical departments underwent surgery for lumbar spinal stenosis with degenerative spondylolisthesis without scoliosis. After excluding patients undergoing laminectomy alone, fusion without instrumentation, or surgery in more than 2 levels and those with a former operation at the index level, 794 patients were included in the analyses, regardless of missing or incomplete follow-up data, before propensity score matching. Data were analyzed from March 20 to October 30, 2018.

**Exposures:**

Microdecompression alone or decompression with instrumented fusion.

**Main Outcomes and Measures:**

A reduction from baseline of 30% or greater in the Oswestry Disability Index at 12-month follow-up.

**Results:**

After propensity score matching, 570 patients (413 female [72%]; mean [SD] age, 64.7 [9.5] years) were included for comparison, with 285 undergoing microdecompression (mean [SD] age, 64.6 [9.8] years; 205 female [72%]) and 285 undergoing decompression with instrumented fusion (mean [SD] age, 64.8 [9.2] years; 208 female [73%]). The proportion of each type of procedure varied between departments. However, changes in outcome scores varied within patients but not between departments. The proportion of patients with improvement in the Oswestry Disability Index of at least 30% was 150 of 219 (68%) in the microdecompression group and 155 of 215 (72%) in the instrumentation group. The 95% CI (–12% to 5%) for the difference of −4% was above the predefined margin of noninferiority (–15%). Microdecompression alone was associated with shorter operation time (mean [SD], 89 [44] vs 180 [65] minutes; *P* < .001) and shorter hospital stay (mean [SD], 2.5 [2.4] vs 6.4 [3.0] days; *P* < .001).

**Conclusions and Relevance:**

Among patients with degenerative spondylolisthesis, the clinical effectiveness of microdecompression alone was noninferior to that of decompression with instrumented fusion. Microdecompression alone was also associated with shorter durations of surgery and hospital stay, supporting the suggestion that the less invasive procedure should be considered for most patients.

## Key Points

**Question:**

How do outcomes compare between microdecompression and decompression with instrumented fusion for patients undergoing surgery for degenerative spondylolisthesis?

**Findings:**

In this comparative effectiveness study including 285 pairs of propensity score–matched patients, 150 of 219 patients (68%) in the microdecompression group and 155 of 215 patients (72%) in the instrumented fusion group achieved an improvement in Oswestry Disability Index of at least 30%, a clinically meaningful noninferior difference. Microdecompression was associated with shorter operation time and shorter length of hospital stay.

**Meaning:**

The findings suggest that microdecompression alone should be considered as an option for most patients undergoing surgery for spinal stenosis with degenerative spondylolisthesis.

## Teaser

This comparative effectiveness study evaluates whether the effectiveness of microdecompression alone is noninferior to decompression with instrumented fusion in a real-world setting among patients with lumbar degenerative spondylolisthesis.

## Introduction

Degenerative spondylolisthesis is a forward slip of one vertebra relative to the vertebra below, occurring in a spondylotic and narrowed spinal segment (ie, lumbar spinal stenosis).^1^ Typical symptoms are low back pain and radiating pain into the buttocks and the legs, especially when standing and walking. The standard surgical procedure has been to decompress the stenosis.^2^ In the early 1990s, 2 landmark studies^3,4^ recommended additional fusion surgery. In the following decades, the rate and complexity of fusion procedures increased dramatically.^5,6^ The fusion rate in the United States more than doubled from 2005 to 2014, and degenerative spondylolisthesis accounted for most fusions.^7^ In 2015, the hospital costs for elective lumbar degenerative fusions exceeded $10 billion, the highest aggregate costs of any surgical procedure in the United States.^8^ Adding instrumented fusion to decompression has been supported by 1 randomized clinical trial (RCT)^9^ and clinical guidelines and reviews.^10,11,12,13^ Another RCT,^14^ registry studies,^15,16^ and systematic reviews^17,18^ have recommended decompression alone.

The role of fusion surgery is controversial,^19,20,21^ and a large surgical practice variation between hospitals is reported. In 2011 to 2013, approximately 50% of patients with degenerative spondylolisthesis in Norway and Sweden underwent fusion procedures^22^ compared with 90% to 95% in other countries.^6,7,22,23^ Industrial boosting with differences in industrial encouragement and lucrative financial reimbursement might explain some of the differences in practice.^24,25^

Only a few small-sample studies^26,27,28,29^ have evaluated the performance of less invasive methods of decompression alone, preserving potentially stabilizing structures of the spine. In this study from the Norwegian Registry for Spine Surgery (NORSpine), we hypothesized that in real-world daily clinical practice, microdecompression alone works as well as decompression with instrumented fusion.

## Methods

### Study Setting, Data Collection, and Patient Selection

The reporting and interpretation of this comparative effectiveness study followed the Strengthening the Reporting of Observational Studies in Epidemiology (STROBE)^30^ recommendations and the International Society for Pharmacoeconomics and Outcomes Research (ISPOR) reporting guideline for cohort studies.^31^ Relative effectiveness was studied using prospectively collected data from NORSpine, a national comprehensive registry for quality control and research. According to NORSpine’s annual report for 2015, the coverage rate for lumbar spine surgery was 93% at the hospital level and 63% at the individual level. The registry receives no funding from industry. At hospital admission (baseline), the patients completed questionnaires, which included patient-reported outcome measures and questions about demographics and lifestyle. The surgeons recorded surgical parameters such as diagnosis, treatment, and occurrence of complications. At the 3- and 12-month follow-up, NORSpine’s central unit sent questionnaires by mail to the patients without the involvement of the surgical units. Written informed consent was obtained from the participants preoperatively, and the Norwegian Committee for Medical and Health Research Ethics Central approved the study.

A total of 1376 patients undergoing surgical procedures for lumbar spinal stenosis with degenerative spondylolisthesis from September 19, 2007, to December 21, 2015, at 35 orthopedic and neurosurgical departments were screened for eligibility. Patients were excluded if they had undergone a previous procedure at the index level(s), a procedure in more than 2 levels, or a procedure with an interspinous device or with an anterior approach. Patients were included regardless of missing or incomplete follow-up data.

The primary and secondary outcomes, the criterion for noninferiority, and the statistical methods were defined before statistical analysis.^32^ Data were analyzed from March 20 to October 30, 2018.

### Treatment Groups

Patients who underwent microdecompression alone had preservation of the midline (ie, the spinous process and the interspinous ligaments), and one of the following techniques was used: (1) unilateral laminotomy, (2) bilateral laminotomy, or (3) unilateral laminotomy and crossover decompression. Magnifying devices (microscopes or loupes) were used. Patients who underwent instrumented fusion had a decompression with or without preservation of the midline structures and with or without visual enhancement and additional posterior pedicle screw instrumentation with or without an intervertebral cage.

### Outcome Measures

The Oswestry Disability Index (ODI), version 2.0^33,34^ is a pain-related disability score of 10 items ranging from 0 (no impairment) to 100 (maximum impairment). The primary outcome was a reduction from baseline of 30% or greater at the 12-month follow-up (ie, a clinically important improvement).^35,36^ A patient achieving this amount of improvement was defined as a responder. Secondary outcome measures included the following.

1. The mean change scores and the mean 12-month follow-up scores for the ODI and the Numeric Rating Scale (NRS), which ranges from 0 (no pain) to 10 (worst pain imaginable) for leg pain and for back pain experienced in the last week;
2. The Global Perceived Effect instrument^37^ with 7 response alternatives, including completely recovered, much improved, slightly improved, unchanged, slightly worse, much worse, and worse than ever, that were trichotomized into substantially improved (completely recovered and much improved), little or no change (slightly improved, unchanged, and slightly worse), and substantially deteriorated (much worse and worse than ever);
3. Duration of surgery and hospital stay;
4. The rate of perioperative complications and adverse events registered on the surgeon form; and
5. The rate of complications and adverse events reported by the patients at the 3-month follow-up.

### Statistical Analysis

To make the distribution of observed baseline patient characteristics in the 2 treatment groups as similar as possible, propensity score matching was performed.^38^ A propensity score, derived from a logistic regression model, is defined as a patient’s baseline probability for receiving decompression plus instrumented fusion, conditional on prespecified plausible confounders (age, sex, American Society of Anesthesiologists grade, body mass index, smoking, ODI, NRS leg pain score, NRS back pain score, the European Quality of Life–5 Dimensions questionnaire, the presence of foraminal stenosis, degenerative disc disease, predominating back pain, number of levels undergoing surgery, and neurological palsy). We used the technique of 1:1 matching without replacement, forming paired cases of microdecompression alone and decompression plus fusion, which had a difference in propensity scores of less than 0.2 in logit of the standard deviation.^38^

The null hypothesis was that the proportion of patients with a clinically important improvement (the responder rate) was at least 15 percentage points lower in the microdecompression group than in the fusion group. The null hypothesis was tested by forming a 2-sided 95% CI for the between-group difference in responder rate and would be rejected if the lower bound of the CI was greater than −15%. A noninferiority margin of −15% was assumed to reflect the advantage of performing microdecompression alone instead of the more extensive and expensive instrumentation.^39,40^ This margin is consistent with analysis in other relevant studies.^39,40,41^ The margin corresponds to a number needed to treat of 7 patients (100/15 [6.67]),^42^ that is, if 7 patients or more need instrumented fusion to achieve 1 additional responder, the cheaper, safer, and less comprehensive method of microdecompression alone should be considered good enough (ie, noninferior).

Level and change in the ODI and NRS leg and back pain scores were estimated by multisample latent growth curve (LGC) models, with full information maximum likelihood^43^ under the assumption of missing at random. Owing to nonlinearity, the models were specified as a latent difference score model, including changes from baseline to 3 months, from 3 to 12 months, and the 12-month follow-up (intercept level). The proportion of each type of procedure varied between departments. Patient data were nested within hospital departments and could show clustering effects within units. However, multilevel analyses showed low interclass correlation values for the ODI of 0.023 (baseline) to 0.042 (12 months), for NRS leg pain of 0.026 (baseline) to 0.067 (3 months), and for NRS back pain of 0.013 (3 months) to 0.066 (12 months). The estimated design effect, taking cluster size and interclass correlation into account, showed the highest value to be 2.00 (leg pain at 3 months), which is in the borderline for nonignorable clustering.^44^ However, multilevel models including random slope variance at the hospital department level showed no department differences in change scores in the 2 intervals.

The intercept variance was found to be statistically significant for NRS leg pain (σ^2^ = 0.49; *P* = .03) but not for the ODI (σ^2^ = 13.52; *P* = .13) or NRS back pain (σ^2^ = 0.12; *P* = .10). Due to this level of clustering and the focus on observations within patients, single-level LGC models were estimated with robust standard errors corrected for clustering with the maximum likelihood robust.^44^

For secondary outcomes, comparisons of treatment groups and corresponding estimates of *P* values and 95% CI were based on 2-sided tests for superiority. SPSS, version 24 (IBM Corporation) was used for descriptive statistics, analyses of continuous variables with 2-sided *t* tests or Mann-Whitney tests depending on the distribution of data, analyses of binary variables with Fisher mid-*P* value and Newcombe hybrid score CIs,^45^ and propensity score matching. The LGC analysis was performed with Mplus 8 (Muthén & Muthén).^46^ *P* < .05 indicated statistical significance.

### Missing Data

A loss to follow-up of about 20% was anticipated.^15,47^ A previous study from NORSpine showed similar clinical outcome measures for compliers and noncompliers,^48^ so longitudinal outcome variables were analyzed under the assumption of missingness at random. An additional missing data LGC analysis under the missing data at random assumption was performed for the matched cohort. Multiple imputation^49^ was used with baseline patient characteristics; several clinical, surgical, and radiological parameters; and outcome variables at baseline and follow-up to generate 70 data sets with complete follow-up scores for the ODI and NRS leg pain and back pain. This procedure is recommended if missing data at random may only be partly assumed.^50^ Including such covariates may increase the probability for missing data at random and reduce the probability of missingness not at random.^50^

### Sample Size

For the primary outcome, choosing a 15% noninferiority margin, a type 1 error of 0.05, and power of 0.90 gave a total sample size of 394. An expected 75% follow-up^15^ rate at 12 months required 263 patients in each group.^51^

## Results

**Figure 1.**
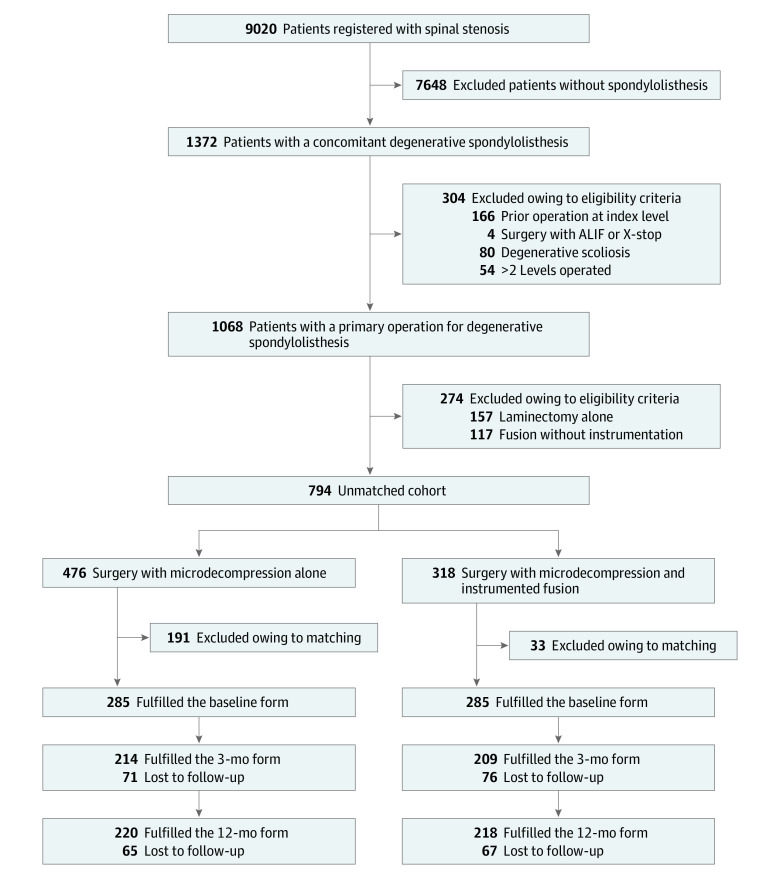
Study Flowchart ALIF indicates anterior lumbar interbody fusion.

Of 794 patients who met study eligible criteria, 476 (60%) underwent microdecompression alone (mean [SD] age, 67.5 [9.9] years; 307 female [64%]), and 318 (40%) underwent decompression plus instrumented fusion (mean [SD] age, 63.5 [10.0] years; 240 female [76%]). After propensity score matching, 570 patients (413 female [72%] and 157 male [28%]; mean [SD] age, 64.7 [9.5] years) remained, of whom 285 patients underwent microdecompression alone (mean [SD] age, 64.6 [9.8] years; 205 female [72%] and 80 male [28%]), and 285 underwent decompression and instrumentation (mean [SD] age, 64.8 [9.2] years; 208 female [73%] and 77 male [27%]). Figure 1 shows the enrollment of participants. Baseline parameters are given in Table 1, and surgical parameters are shown in eTable 1 in the Supplement.

**Table 1.** Baseline Data for the Unmatched and Matched Cohorts

Characteristic	Unmatched cohort			Propensity score–matched cohort		
	Microdecompression alone (n = 476)	Decompression plus instrumented fusion (n = 318)	*P* value	Microdecompression alone (n = 285)	Decompression plus instrumented fusion (n = 285)	*P* value
Age, mean (SD), y^a^	67.5 (9.9)	63.5 (10.0)	<.001	64.6 (9.8)	64.8 (9.2)	.79
Female sex, No./total No. (%)^a^	307/476 (65)	240/318 (75)	<.001	205/285 (72)	208/285 (73)	.78
≥3 y of Education, No./total No. (%)^a^	149/464 (32)	104/314 (33)	.77	98/280 (35)	93/281 (33)	.64
ASA score, mean (SD)^b^^,^^c^	2.10 (0.58)	1.97 (0.52)	<.001	2.01 (0.58)	2.00 (0.51)	.94
BMI, mean (SD)^a^	26.9 (4.4)	27.1 (5.2)	.65	27.1 (4.4)	26.7 (4.6)	.41
Smoker, No./total No. (%)^a^	81/474 (17)	56/315 (18)	.80	57/283 (20)	51/282 (18)	.54
Anxiety or depression, No./total No. (%)^a^^,^^d^	165/463 (36)	131/303 (43)	.04	112/280 (40)	109/271 (40)	.96
Disc degeneration, No./total No. (%)^c^	103/476 (22)	52/318 (16)	.07	47/285 (16)	49/285 (17)	.82
Foraminal stenosis, No./total No. (%)^c^	40/476 (8)	35/318 (11)	.22	28/285 (10)	27/285 (9)	.89
Leg pain >1 y, No./total No. (%)^a^	303/438 (69)	211/295 (72)	.50	194/266 (73)	184/265 (69)	.37
Back pain >1 y, No./total No. (%)^a^	348/446 (78)	254/303 (84)	.50	224/268 (84)	222/270 (82)	.68
Use of analgesics, No./total No. (%)^a^	369/469 (79)	272/316 (86)	.009	238/282 (84)	239/283 (84)	.99
Neurological palsy, No./total No. (%)^c^	30/476 (6)	14/318 (4)	.25	14/285 (5)	13/285 (5)	.84
Predominant back pain, No./total No. (%)^a^	28/407 (7)	25/264 (9)	.23	23/241 (10)	22/237 (9)	.92
ODI, mean (SD)^a^^,^^e^	40.2 (15.4)	41.4 (14.4)	.30	41.3 (15.6)	40.8 (14.1)	.69
NRS leg pain, mean (SD)^a^^,^^f^	6.9 (2.1)	6.6 (2.1)	.12	6.7 (2.1)	6.7 (2.0)	.97
NRS back pain, mean (SD)^a^^,^^f^	6.7 (2.1)	6.8 (1.9)	.81	6.7 (2.0)	6.8 (1.9)	.70

Abbreviations: ASA, American Society of Anesthesiologists; BMI, body mass index (calculated as weight in kilograms divided by height in meters squared); NRS, Numeric Rating Scale; ODI, Oswestry Disability Index.

^a^ Data collected from patient forms.

^b^ Scores range from 1 (no presence of disease) to 4 (life-threatening disease).

^c^ Data collected from surgeon forms.

^d^ Includes patients who replied “moderately anxious or depressed” or “extremely anxious or depressed” according to the European Quality of Life–5 Dimensions questionnaire.

^e^ Scores range from 0 to 100, with higher scores indicating more disability.

^f^ Scores range from 0 to 10, with higher scores indicating more pain.

### Unmatched Cohort

**Figure 2.**
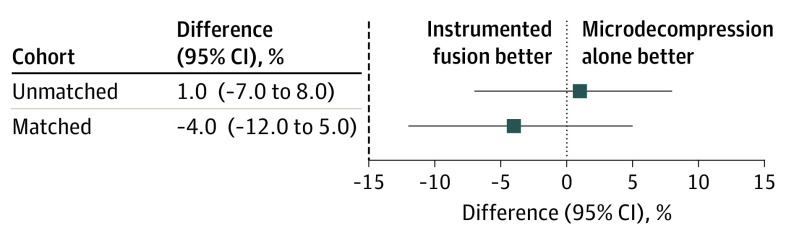
Differences Between Treatment Groups in Primary Outcome Data show the proportion of patients with reduction from baseline of 30% or greater in the Oswestry Disability Index at the 12-month follow-up. Dashed line indicates the predefined margin of noninferiority.

Follow-up scores of the ODI and NRS leg pain and back pain are shown in eTable 2 in the Supplement. The proportion of patients with a clinically important improvement of the ODI at 12 months was 243 of 343 (71%) in the microdecompression group and 163 of 232 (70%) in the instrumentation group (difference, 1%; 95 CI, –7% to 8%) (Figure 2).

### Propensity Score–Matched Cohort

Table 1 demonstrates that propensity score matching created similar groups with respect to the distribution of observed baseline parameters. The follow-up rate was 423 of 570 (74%) at 3 months and 438 of 570 (77%) at 12 months (Figure 1), and 479 of 570 participants (84%) had at least 1 follow-up registration.

### Primary Outcome

The proportion of patients with a clinically important improvement in the ODI at the 12-month follow-up was 150 of 219 (68%) in the microdecompression group and 155 of 215 (72%) in the instrumented fusion group. The lower bound of the 95% CI (–12% to 5%) for the between-group difference of −4% did not cross the −15% limit of noninferiority (Figure 2). An absolute difference of 4% corresponds to a number needed to treat of 25 patients (95% CI, 8 to ∞).

### Secondary Outcomes

Table 2 shows the mean change from baseline to 3 months, the mean change from 3 to 12 months, and the mean 12-month follow-up scores for the ODI and NRS leg and back pain. At 12 months, we observed no statistically significant difference in mean ODI scores between microdecompression alone and instrumented fusion (mean [SD], 22.2 [18.2] and 20.5 [17.7], respectively; mean difference 1.7 [95% CI, –2.4 to 5.8]; *P* = .42). At 12 months, the microdecompression group had statistically significantly higher scores for NRS leg pain (mean [SD], 3.5 [3.0] vs 2.7 [2.9]; mean difference, 0.8 [95% CI, 0.1-1.4]; *P* = .02) and NRS back pain (mean [SD], 3.8 [2.9] vs 3.3 [2.6]; mean difference, 0.6 [95% CI, 0.01-1.1]; *P* = .04) compared with the instrumented fusion group.

**Table 2.** Patient-Reported Outcomes in the Propensity Score–Matched Cohort, Estimated With Latent Growth Curve Models

Variable	Mean (SD) score^a^		Difference^b^	
	Microdecompression alone (n = 285)	Decompression plus instrumented fusion (n = 285)	Mean (95% CI)	*P* value
ODI^c^				
Change 0 to 3 mo	–20.0 (17.6)	–19.4 (18.4)	–0.6 (–4.5 to 3.3)	.77
Change >3 to 12 mo	0.9 (12.7)	–0.9 (14.4)	1.8 (–1.3 to 4.8)	.26
12-mo Follow-up	22.2 (18.2)	20.5 (17.7)	1.7 (–2.4 to 5.8)	.42
NRS leg pain^d^				
Change 0 to 3 mo	–3.7 (3.2)	–4.4 (3.3)	0.7 (0.2 to 1.2)	.01
Change >3 to 12 mo	0.5 (2.6)	0.4 (2.6)	0.1 (–0.4 to 0.6)	.69
12-mo Follow-up	3.5 (3.0)	2.7 (2.9)	0.8 (0.1 to 1.4)	.02
NRS back pain^d^				
Change 0 to 3 mo	–3.3 (2.9)	–3.6 (2.9)	0.3 (–0.3 to 0.9)	.33
Change >3 to 12 mo	0.4 (2.5)	0.06 (2.6)	0.3 (–0.2 to 0.9)	.22
12-mo Follow-up	3.8 (2.9)	3.3 (2.6)	0.6 (0.01 to 1.1)	.04

Abbreviations: NRS, Numeric Rating Scale; ODI, Oswestry Disability Index.

^a^ Estimated with multisample latent growth curve models.

^b^ Calculated as the score for microdecompression alone minus the score for decompression and instrumented fusion with 95% CIs and 2-sided tests for superiority.

^c^ Scores range from 0 to 100, with higher scores indicating more disability.

^d^ Scores range from 0 to 10, with higher scores indicating more pain.

According to the Global Perceived Effect at the 12-month follow-up (eTable 3 in the Supplement), the rate of substantial improvement (145 of 218 [67%] for microdecompression and 154 of 217 [71%] for fusion; difference, –4% [95% CI, –13% to 4%]; *P* = .32) and the rate of substantial deterioration (9 of 218 [4%] for microdecompression and 8 of 217 [4%] for fusion; difference, 0 [95% CI, –3% to 4%]; *P* = .81) did not differ between groups.

The duration of surgery (mean [SD], 89 [44] vs 180 [65] minutes; difference, –91 [95% CI, –100 to –81] minutes; *P* < .001) and the length of hospital stay (mean [SD], 2.5 [2.4] vs 6.4 [3.0] days; difference, −3.9 [95% CI, –4.4 to –3.4] days; *P* < .001) was statistically significantly shorter for microdecompression alone than for instrumented fusion (Table 3). The microdecompression group had fewer surgeon-reported perioperative complications than the fusion group (7 of 285 [2%] vs 24 of 285 [8%]; difference, –6% [95% CI, –10% to –2%]; *P* = .003). In both groups, the most common complication was a dural tear (5 of 285 [2%] and 16 of 285 [6%]; difference, –4% [95% CI, –7% to 0%]; *P* = .02). Patients undergoing microdecompression alone reported a statistically significantly higher incidence of superficial wound infection than the fusion group during the first 3 months postoperatively (16 of 209 [8%] and 5 of 174 [3%]; difference, 5% [95% CI, 0 to 10%]; *P* = .04). Other registered complications are listed in Table 3.

**Table 3.** Operation Time, Length of Hospital Stay, and Complications for the Propensity Score–Matched Cohort

Variable	Microdecompression alone (n = 285)	Decompression plus instrumented fusion (n = 285)	Difference (95% CI)^a^	*P* value^b^
Operation time, mean (SD), min^c^	89 (44)	180 (65)	–91 (–100 to –81)	<.001
Duration of hospital stay, mean (SD), d^c^	2.5 (2.4)	6.4 (3.0)	–3.9 (–4.4 to –3.4)	<.001
Perioperative complications, No./total No. (%)^d^	7/285 (2)	24/285 (8)	–6 (–10 to –2)	.003
Dural tears	5/285 (2)	16/285 (6)	–4 (–7 to 0)	.02
Nerve root lesion	0	1/285 (0.4)	NC	NC
Operation on wrong side/level	1/285 (0.4)	1/285 (0.4)	NC	NC
Blood transfusion	1/285 (0.4)	1/285 (0.4)	NC	NC
Misplaced implants	NA	2/285 (1)	NC	NC
Cardiac complication	1/285 (0.4)	1/285 (0.4)	NC	NC
Patient-reported complications during the first 3 mo, No./total No. (%)^e^				
Superficial wound infection	16/209 (8)	5/174 (3)	5 (0 to 10)	.04
Deep wound infection	3/207 (1)	0/174	NC	NC
Deep venous thrombosis	1/207 (0.5)	0/174	NC	NC
Lung thrombosis	1/207 (0.5)	2/174 (1)	NC	NC
Pneumonia	5/207 (2)	2/174 (1)	NC	NC
Urinary tract infection	17/207 (8)	14/174 (8)	0 (–5 to 6)	.96

Abbreviations: NA, not applicable; NC, not computed.

^a^ Calculated as microdecompression alone minus decompression and fusion with 95% CI.

^b^ Calculated using 2-sided tests for superiority.

^c^ Collected during hospital stay.

^d^ Collected from surgeon forms.

^e^ Collected from patient forms.

eTable 4 in the Supplement shows 12-month follow-up results for the LGC models subsequent to multiple imputation of missing data. At 12 months, there were no differences in the ODI and NRS back pain between the groups, whereas NRS leg pain was statistically significantly higher for the microdecompression group than for the instrumented fusion group (mean [SD], 3.5 [3.0] and 2.8 [3.0]; mean difference, 0.7 [95% CI, 0.1-1.3]; *P* = .03).

## Discussion

Microdecompression alone was noninferior to decompression plus instrumented fusion. The result of the primary outcome was supported by the patients’ global perceived effects and by analyses of the mean ODI scores both before and after imputation of missing data. Furthermore, microdecompression alone was associated with considerably shorter duration of surgery and hospital stay and somewhat fewer surgeon-reported perioperative complications. Patients treated with instrumented fusion had slightly less leg and back pain and fewer patient-reported superficial wound infections.

Other unmatched observational studies^26,27,28,29^ have found nondifferent outcomes between microdecompression alone and decompression plus instrumented fusion. Unlike our study, these studies did not reveal any between-group differences in outcome scores for leg or back pain.

Following 2 simultaneously published RCTs,^9,14^ the role of fusion has been debated.^19,20,21,52,53^ In the RCT by Ghogawala et al,^9^ 51 of 71 patients underwent procedures performed by 1 surgeon. Decompression alone resulted in less improvement in the physical component of the generic 36-Item Short Form Health Survey than instrumented fusion 2 years after surgery. The improvement in ODI scores was not statistically significantly different between the groups. The more pragmatic multicenter RCT by Försth et al^14^ (n = 135) used the ODI as the primary outcome measurement and revealed baseline scores as well as follow-up scores in accordance with our results.

Randomized clinical trials are the criterion standard for studying treatment efficacy, but their generalizability has been questioned owing to strictly recruited patients and clinicians and enforced treatment allocation.^54^ This study was designed to reflect real-world relative effectiveness between carefully matched groups. The aim was to study patients recruited in daily clinical practice at several different hospitals and the treatments chosen according to surgeon and patient preferences.^54,55^ Our study provides knowledge about how treatments work in the more pragmatic delivery of health care.^54,56,57^

Based on the present study as well as previous pragmatic studies^14,16^ and considering the large clinical practice variation,^6,22,23^ the high rate of instrumented fusion seems unreasonable. In accordance with a priori expectations and former studies,^23,58^ our findings of shorter operation times and hospital stays indicate that a microdecompression alone is associated with acceptable clinical results at lower costs. Although instrumented fusion was associated with somewhat more pain reduction, the high number needed to achieve 1 additional responder and the somewhat higher perioperative complication rate showed disadvantages of instrumentation. Altogether, we consider the noninferior clinical effectiveness and the potential health-economic benefits of microdecompression alone to surpass the procedure’s potential inferiority.

However, this study does not provide evidence that microdecompression alone should be the preferred method for all patients. Adding fusion to decompression may still be a good treatment option for subgroups. Owing to a lack of evidence for defining such subgroups, future research should endeavor to identify variables associated with the best treatment for each individual.^39^

### Limitations

The diagnoses of spinal stenosis and degenerative spondylolisthesis are based on the surgeons’ evaluation of radiographs, the radiological descriptions, and clinical signs and symptoms. We have not retrospectively checked whether all established diagnostic criteria^59^ were fulfilled. Incorrect diagnoses may therefore have been undetected. Furthermore, data on reoperations and data beyond the 12-month follow-up are lacking. Some studies have found lower reoperation rates when a decompression has been supported by fusion,^9,60^ whereas other studies have demonstrated similar^14^ and even higher^27^ reoperation rates after an additional fusion. For a mixed population undergoing spinal surgical procedures, the clinical outcomes at the 12-month follow-up seem to be the same as the 2-year outcomes^61,62^ and stable even at the 5-year follow-up.^26,63,64^

Finally, it is important to recognize that, unlike an RCT, this study was not able to detect treatment-related differences in efficacy. Although the propensity score matching equalized the baseline scores regarding the observed parameters, the distribution of unobserved parameters (eg, radiological parameters and potential differences associated with patients’ expectations owing to given information) might have been unbalanced. A risk of residual bias does therefore still exist.

## Conclusions

This study found that microdecompression alone seems to be not appreciably worse than decompression and instrumented fusion for treatment of degenerative spondylolisthesis. We would carefully suggest the less extensive and less expensive treatment as the primary surgical choice for most patients with lumbar degenerative spondylolisthesis.

## Notes

Supplement. **eTable 1.** Surgical Parameters for the Unmatched Cohort and for the Propensity Score–Matched Cohort **eTable 2.** Outcome Scores for the Unmatched Cohort **eTable 3.** Responses on the Global Perceived Effect Scale for the Propensity Score–Matched Cohort **eTable 4.** Outcomes for the Propensity Score–Matched Cohort Following Multiple Imputation of Missing Data
