# Supplementary material for: Comparative Effectiveness of Microdecompression Alone vs Decompression Plus Instrumented Fusion in Lumbar Degenerative Spondylolisthesis
Source: JAMA Netw Open. 2020 Sep 10;3(9):e2015015. doi: 10.1001/jamanetworkopen.2020.15015 (PMC7489859; doi:10.1001/jamanetworkopen.2020.15015)
Supplement: Supplement. — eTable 1. Surgical Parameters for the Unmatched Cohort and for the Propensity Score–Matched Cohort eTable 2. Outcome Scores for the Unmatched Cohort eTable 3. Responses on the Global Perceived Effect Scale for the Propensity Score–Matched Cohort eTable 4. Outcomes for the Propensity Score–Matched Cohort Following Multiple Imputation of Missing Data [file jamanetwopen-e2015015-s001.pdf]

Supplementary Online Content

Austevoll IM, Gjestad R, Solberg T, et al. Comparative effectiveness of microdecompression alone vs decompression plus instrumented fusion in lumbar degenerative spondylolisthesis. *JAMA Netw Open*. 2020;3(9):e2015015. doi:10.1001/jamanetworkopen.2020.15015

eTable 1. Surgical Parameters for the Unmatched Cohort and for the Propensity Score–Matched Cohort

eTable 2. Outcome Scores for the Unmatched Cohort

eTable 3. Responses on the Global Perceived Effect Scale for the Propensity Score–Matched Cohort

eTable 4. Outcomes for the Propensity Score–Matched Cohort Following Multiple Imputation of Missing Data

This supplementary material has been provided by the authors to give readers additional information about their work.

eTable 1. Surgical Parameters for the Unmatched Cohort and for the Propensity Score–Matched Cohort

	Unmatched cohort		Propensity score matched cohort	
	Microdecompression alone N=476	Decompression and instrumented fusion N=318	Microdecompression alone N=285	Decompression and instrumented fusion N=285
	No. (%)			
Operated with magnifying devices	476 (100)	211 (66)	285 (100)	183 (64)
Operated on level L2/L3	15 (3)	9 (3)	7 (3)	9 (3)
Operated on level L3/L4	142 (30)	89 (38)	88 (31)	83 (29)
Operated on level L4/L5	387 (81)	261 (82)	235 (82)	235 (82)
Operated on level L5/S1	34 (7)	31 (10)	25 (9)	23 (8)
One level operated	372 (78)	240 (75)	214 (75)	216 (76)
Two levels operated	104 (22)	78 (25)	71 (25)	69 (24)
Laminectomy	0	201 (63)	0	180 (63)
Midline preserving decompression	476 (100)	117 (37)	285 (100)	105 (37)
-unilateral decompression	86 (18)	17 (5)	58 (20)	14 (05)
-bilateral decompression	306 (64)	31 (10)	188 (66)	26 (9)
-bilateral decompression, unilateral approach	84 (18)	69 (2)	39 (14)	65 (23)
Posterolateral instrumented fusion without cage	0	205 (64)	0	189 (66)
Posterolateral interbody fusion (PLIF)	0	9 (3)	0	9 (3)
Transforaminal interbody fusion (TLIF)	0	104 (33)	0	87 (31)

Abbreviation: N, number of patients with complete data

All data collected from surgeon forms. **Numbers (%) of total (N) are given.**

©2020 Austevoll IM et al. *JAMA Network Open*.

eTable 2. Outcome Scores for the Unmatched Cohort

		Micro-decompression alone		Decompression and instrumented fusion			
		N	Mean (SD)	N	Mean (SD)	Difference (95% CI)[a]	*P* value[a]
ODI	3 months	351	20.1 (18.2)	230	21.7 (16.0)	−1.6 (−4.5 to 1.3)	.28
	12 months	346	20.6 (17.9)	224	21.0 (17.7)	−0.5 (−3.4 to 2.5)	.76
NRS leg pain	3 months	346	2.9 (2.9)	220	2.2 (2.6)	0.7 (−03 to1.2)	.002
	12 months	343	3.4 (3.1)	228	2.8 (2.9)	0.6 (0.1 to 1.1)	.02
NRS back pain	3 months	348	3.3 (2.7)	224	3.3 (2.4)	0.1 (−0.4 to 0.5)	.76
	12 months	348	3.6 (2.9)	233	3.4 (2.6)	0.2 (−0.2 to 0.7)	.32

Abbreviations: SD, standard deviation; N, number of patients for analysis; CI, confidence interval; ODI, Oswestry Disability Index (ranges from 0 to 100, with higher scores indicating more disability); NRS, Numeric rating scale (ranges from 0 to 10, with higher scores indicating more pain).

[a]Two-sided tests for superiority. Unadjusted analyses.

©2020 Austevoll IM et al. *JAMA Network Open*.

eTable 3. Responses on the Global Perceived Effect Scale for the Propensity Score–Matched Cohort

	**Micro-decompression alone (N=218)**	**Decompression and instr. fusion (N=217)**	Difference (95% CI)[c]	*P* value[d]
Replies	No. (%)			
"Completely recovered"	51 (23)	49 (23)		.72
"Much improved"	94 (43)	105 (48)		
"Slightly improved"	45 (21)	43(20)		
"Unchanged"	12 (5.5)	5 (2.3)		
"Slightly worse"	7 (3.2)	7 (3.2)		
"Much worse"	5 (2.3)	5 (2.3)		
"Worse than ever"	4 (1.8)	3 (1.4)		
Substantially improved[a]	145 (67)	154 (71)	−4% (−13 to 4)	.32
Substantially deteriorated[b]	9 (4)	8 (4)	0% (−3 to 4)	.81

Abbreviations: N, number of patients completed the **Global Perceived Effect questionnaire at 12 months**.

[a] Patients rated themselves as 'completely recovered' or 'much improved'.

[b] Patients rated themselves as 'much worse' or 'worse than ever'.

[c] The absolute difference (microdecompression alone minus decompression and instrumented fusion).

[d] Pearson's Chi-square test

©2020 Austevoll IM et al. *JAMA Network Open*.

eTable 4. Outcomes for the Propensity Score–Matched Cohort Following Multiple Imputation of Missing Data

	Micro-decompression alone (N=285)	Decompression and instrumented fusion (N=285)	Difference[a]	
	Mean (SD)	Mean (SD)	Mean (95% CI)	*P* value
ODI				
Change 0-3 months	−20.0 (17.6)	−19.2 (18.4)	−0.9 (−4.6 to 2.9)	.65
Change >3-12 months	0.73 (12.7)	−0.9 (14.4)	1.6 (−1.3 to 4.5)	.28
12-month follow-up	22.0 (18.2)	20.8 (17.7)	1.2 (−2.6 to 5.0)	.53
NRS leg pain				
Change 0-3 months	−3.7 (3.2)	−4.4 (3.3)	0.7 (0.1 to 1.3)	.02
Change >3-12 months	0.4 (2.7)	0.5 (2.7)	−0.03 (−0.6 to 0.5)	.91
12-month follow-up	3.5 (3.0)	2.8 (3.0)	0.7 (0.1 to 1.4)	.03
NRS back pain				
Change 0-3 months	−3.3 (2.9)	−3.5 (2.9)	0.3 (−0.4 to 0.9)	.43
Change >3-12 months	0.4 (2.5)	0.1 (2.6)	0.3 (−0.2 to 0.8)	.25
12-month follow-up	3.9 (2.9)	3.4 (2.6)	0.5 (−0.07 to 1.0)	.08

The mean change scores (SD) from baseline to 3-month follow-up and from 3- to 12-month follow-up (negative values indicate improvement) and the mean follow-up scores (SD) at 12-month follow-up, estimated with multi-sample Latent Growth Curve models.

Abbreviations: SD, standard deviation; N, number of patients for analysis; CI, confidence interval; ODI, Oswestry Disability Index (ranges from 0 to 100, with higher scores indicating more disability), NRS, Numeric rating scale (ranges from 0 to 10, with higher scores indicating more pain).

[a]Microdecompression alone minus decompression and instrumented fusion. 95% CIs and 2-sided tests for superiority.

©2020 Austevoll IM et al. *JAMA Network Open*.
